# Inhaled delivery of 23-valent pneumococcal polysaccharide vaccine does not result in enhanced pulmonary mucosal immunoglobulin responses

**DOI:** 10.1016/j.vaccine.2008.07.082

**Published:** 2008-10-03

**Authors:** Stephen B. Gordon, Rose Malamba, Neema Mthunthama, Elizabeth R. Jarman, Kondwani Jambo, Khuzwayo Jere, Eduard E. Zijlstra, Malcolm E. Molyneux, John Dennis, Neil French

**Affiliations:** a Pulmonary Immunology, Liverpool School of Tropical Medicine, Pembroke Place, Liverpool L3 5QA, United Kingdom; b Malawi-Liverpool-Wellcome Programme of Clinical Tropical Research, Universities of Malawi and Liverpool (UK), Queen Elizabeth Central Hospital, PO Box 30096, Chichiri, Blantyre, Malawi; c Department of Medicine, University of Malawi College of Medicine, PO Box 360, Blantyre, Malawi; d Department of Community Health Sciences, Faculty of Medicine, University of Calgary, 3330 Hospital Drive NW Calgary, Alberta T2N 4N1, Canada; e Karonga Prevention Programme, Chilumpha, Malawi and London School of Hygiene and Tropical Medicine, Keppel Street, London, United Kingdom

**Keywords:** Inhaled pneumococcal vaccine, Lung, Pneumonia, *Streptococcus pneumoniae*, Immunoglobulin, Mucosa, Bronchoalveolar lavage, Polysaccharide

## Abstract

We compared the effect of intramuscular vs. inhaled 23-valent pneumococcal capsular polysaccharide vaccine (23-PPV) on pulmonary mucosal immunoglobulin levels. Bronchoalveolar lavage (BAL) and serum were collected from 33 adults before and 1 month after injected (*n* = 16) or inhaled (*n* = 17) 23-PPV. Levels of pneumococcal capsule-specific IgG and IgA to types 1, 9V and 14 were measured in each sample. Injected 23-PPV produced a significant increase in types 1, 9V and 14 capsule-specific IgG and type 1 IgA in both serum and BAL (type 1 geometric mean BAL IgG 9.8 ng/ml post-vaccine vs. 5 ng/ml pre-vaccine, *p* = 0.01; type 9V geo mean 5.6 ng/ml vs. 2.7 ng/ml, *p* = 0.001; type 14 geo mean 23.6 ng/ml vs. 6.2 ng/ml, *p* = 0.02). Inhaled vaccine produced no response in either BAL or serum.

## 1 Introduction

*Streptococcus pneumoniae* is the most common cause of pneumonia world-wide [1] as well as being a major cause of invasive disease syndromes, such as bacteraemia and meningitis, and mucosal syndromes such as otitis media and sinusitis [2]. The burden of pneumonia [3] and bacterial meningitis [4] are particularly high in Africa, where the incidence and severity of these infections is greatly increased in young children and among patients also infected with the human immunodeficiency virus (HIV) [5,6]. Mucosal syndromes are common in developed nations where otitis media is a particular problem among young children in day-care and pneumonia is a common cause of death in the elderly.

Vaccination against pneumococcal infection has been an important goal for many years with much effort focused on the pneumococcal polysaccharide capsule which is a major virulence factor for the bacteria [7]. Pneumococcal carriage and disease are known to induce host immunity against recurrent infection with the same capsular type (90 types have been described) both in immunocompetent adults and HIV infected adults [8]. This immunity has been assumed to be due to the production of opsonic antibody specific to the pneumococcal capsular polysaccharide because early passive transfer experiments demonstrated protection in animal experiments and serum therapy was used as a therapy for pneumococcal disease in the pre-antibiotic era [9]. Increased levels of capsule-specific antibody have been noted in both serum and lung fluid following pneumococcal disease in both immunocompetent and HIV infected adults [10]. The first pneumococcal vaccine trials used whole dead bacteria [11] quickly followed by purified capsular polysaccharide. This induced anti-capsular antibody, predominantly IgG, and serum levels of immunoglobulin have been used as a surrogate measure of clinical protection. The first pneumococcal polysaccharide vaccines were effective at reducing disease in groups at high risk of transmission, such as mine workers [12] or military recruits, but were ineffective in young children [13] and HIV infected adults [14]. In addition, it was noted that not only was the 23-valent pneumococcal polysaccharide vaccine (23-PPV) ineffective at preventing pneumonia in adults, but that it actually caused an excess of pneumonia in several randomized trials [15]. This excess of pneumonia reached statistical significance in trials involving both HIV infected adults [14] and elderly people [16]. The 23-PPV vaccine continues to be recommended in the elderly and other adult groups at risk of pneumococcal disease, however, as it does offer protection against invasive pneumococcal disease [17].

The paradox surrounding pneumonia in 23-PPV vaccination was recently overshadowed by the major success of the 7-valent pneumococcal conjugate vaccine (7-PCV) which has already resulted in an important reduction in pneumococcal disease in the USA [18]. A similar 9-valent vaccine has been shown to prevent pneumococcal infections in both HIV infected and healthy children in South Africa [19] and the Gambia [20], and has shown a significant reduction in all-cause mortality in vaccinated children in the Gambia study. Conjugate vaccines are also substantially less effective, however, at preventing pneumonia than invasive pneumococcal disease [21]. The prevention of pneumonia is an important priority in pneumococcal vaccination as the number of cases of pneumonia is more than double the combined total of bacteraemia and meningitis [18].

We have addressed the hypothesis that vaccination may differ from native disease in that vaccination may fail to induce adequate levels of protective pulmonary mucosal immunoglobulin. Pulmonary humoral responses are locally regulated as illustrated by the poor correlation between pulmonary and systemic levels of cellular inflammation [22] and immunoglobulin response [23] to both inhaled antigen and pulmonary infection. We showed that specific anti-pneumococcal capsular polysaccharide responses in lung fluid following pneumococcal infection were regulated independently of responses seen in the serum, and that these responses were preserved in HIV infected subjects [10]. Further, we also showed that although conjugate vaccination resulted in increased pneumococcal capsule-specific immunoglobulin in lung fluid from both HIV infected and immunocompetent adults [24], the immunoglobulin function was impaired in HIV infected subjects [25].

Two recent studies have shown that inhaled 23-PPV resulted in a detectable serum response in healthy volunteers [26] and patients with chronic obstructive pulmonary disease [27] but pulmonary responses were not measured. We compared the pulmonary mucosal responses to subcutaneous and inhaled 23-PPV to determine if the inhaled route offered any advantage in protecting the mucosal surface.

## 2 Methods

### 2.1 Subject recruitment and selection

Adult volunteers with no history of pneumonia or asthma and a normal chest X-ray were recruited by advertisement in the Queen Elizabeth Central Hospital, Malawi. All volunteers gave written informed consent to HIV testing, histamine challenge test and participation in a single-blind trial of injected vs. inhaled 23-valent pneumococcal polysaccharide vaccine.

Initially, volunteers consented to HIV testing. We wished to exclude HIV positive volunteers as the 23-valent pneumococcal polysaccharide vaccine (23-PPV) has been shown in a double-blind randomized controlled trial to be harmful in HIV-infected subjects [14]. HIV negative volunteers carried out a modified histamine challenge test to exclude subjects with latent asthma [28]. Subjects inhaled increasing numbers of breaths of saline and then 8 mg/ml histamine from 1 breath to a maximum of 32 breaths with 3 forced expiration in one second (FEV1) blowing tests after each dose. The test was discontinued if the volunteer experienced either respiratory symptoms or a 15% reduction from baseline FEV1. This test excluded mild asthma patients after 4–8 breaths of histamine; all subjects proceeding to the vaccine study were able to inhale 32 breaths of 8 mg/ml histamine without a 15% drop in FEV1.

This study was given ethical approval by the Liverpool School of Tropical Medicine Research Ethics Committee and the College of Medicine Research Ethics Committee of the University of Malawi.

### 2.2 Bronchoscopy and bronchoalveolar lavage (BAL)

Bronchoscopy and bronchoalveolar lavage was carried out at baseline and at 1 month after vaccination on all subjects and serum was stored at each visit. Lavage samples (BAL) were collected as previously described [29]. Briefly, topical lignocaine was applied to nasal and pharyngeal mucosa in semi-recumbent subjects. A fiber-optic bronchoscope (Olympus, UK) was passed to the level of a sub-segmental bronchus of the right middle lobe and four 50 ml aliquots of sterile normal saline at 37 °C instilled and removed using gentle hand suction. The aspirated bronchoalveolar lavage fluid was placed into siliconised glass containers and transferred immediately to the laboratory for processing within 30 min. BAL samples were spun to obtain a cell pellet and the supernatant fluid stored in multiple aliquots at −80 °C for subsequent analysis by ELISA.

### 2.3 Allocation to subcutaneous or inhaled vaccine (23-PPV)

Subjects listed by date of recruitment in separate gender lists were alternately allocated either inhaled or subcutaneous vaccine. All subjects and clinic staff were unaware of the allocation of any subject, and all subjects received both inhalations and an injection. Subjects randomized to receive subcutaneous vaccine received 3 ml of nebulised saline by mouth inhalation on days 1 and 2 and a 0.5 ml 23-PPV injection was given subcutaneously on day 2. Subjects randomized to receive inhaled vaccine received 3 ml nebulised saline containing 50 μl 23-PPV (1/10 of a vaccine) by oral inhalation on day 1 and 3 ml of nebulised saline containing 0.5 ml 23-PPV (a whole vaccine) by oral inhalation on day 2. A sham injection of saline was also given subcutaneously on day 2. The technicians carrying out the inhalations and FEV1 tests, and the nurses giving the injections and recording side-effects were unaware of whether the inhalations or injection contained active vaccine for each patient. All patients carried out check FEV1 measurements 10, 30, 60 and 180 min after all inhalations.

### 2.4 Nebulised delivery of 23-PPV

A simple jet nebuliser was selected (HS860 reuseable Sidestream, Respironics, USA) to nebulise the vaccine as more complex piezo-electric systems (MicroAir, Omron, UK) became blocked by the vaccine. The same nebuliser cup and source of pressurized air was used for all participants [30]. The size of the inhaled particles generated by the nebuliser expressed as the median mass aerosol diameter (MMAD) for 23-PPV in this nebuliser was measured using a low-flow cascade impactor in two complete trials using sodium fluoride impaction on to filters. Separately, the integrity of the vaccine post-nebuliser was confirmed by condensing a nebulised vaccine and measuring the 23-PPV concentration by sandwich ELISA. Briefly, rabbit type1 serum (Statens Seruminstitut, Denmark) was used as the capture antibody, with pooled human immune serum as the detection antibody and alkaline phosphatase conjugated goat anti-human IgG as the secondary antibody. After checker-board confirmation of optimal ELISA conditions, 23-PPV solution and condensed post-nebuliser aerosol were compared at eight serial dilutions. Finally, the distribution of the vaccine between the eight different nebulised fractions in the cascade impactor was compared by measuring the concentration of 23-PPV eluted from impact filters.

### 2.5 ELISA measurement of pneumococcal capsule-specific IgG and IgA

All samples for ELISA measurement of pneumococcal capsule-specific IgG and IgA were pre-adsorbed using CPS and 22f adsorption steps [31] in order to remove non-functional immunoglobulin. A capsular polysaccharide-specific capture ELISA was carried out using type 1, type 9V and type 14 capsular polysaccharide to measure vaccine-specific responses (American Type Culture Collection (ATCC), Manassas, USA). These are strains that elicit a range of antibody responses following natural exposure and commonly cause disease in our area. Alkaline phosphatase conjugated anti-human IgG or IgA (Sigma, UK) were used as secondary antibodies and Pneumococcal Standard Serum, Lot 89-S (Center for Biological Evaluation and Review, Food and Drug Administration, Bethesda, MD; kindly donated by Dr Carl Frasch) was used as the standard. All samples were run in triplicate in four dilutions, and samples with a CV of greater than 15% were repeated.

### 2.6 Sample size and data analysis

The study design assumed that a threefold increase in serum levels of capsule-specific immunoglobulin level would be measured in response to vaccine in all subjects, and that the range of values obtained would be similar to those in earlier studies [10]. Using these assumptions, a sample size of 15 subjects per group was predicted to be sufficient to have more than 99% power to detect a difference in post-vaccination immunoglobulin level at the 5% significance level. We planned to recruit 17 subjects to each group to allow for voluntary subject withdrawal from the study prior to the second bronchoscopy.

Clinical and experimental data were entered and checked using an anonymised Microsoft Access database, and transferred to Stata version 8 (Statacorp, USA) for analysis. Data were initially plotted by test group (subcutaneous vs. inhaled vaccine) using box plots. Baseline values were confirmed to be matched by non-paired *t*-tests on log transformed data (if Shapiro Wilks testing showed that log transformed data did not follow a normal distribution, the two-sample Wilcoxon test was used). Levels of capsule-specific IgG and IgA for three serotypes were then compared within subject using paired *t*-test on log transformed data to test for a significant effect of vaccination in both serum and BAL. If log transformed data did not form a normal distribution, paired comparisons of before and after vaccination levels of immunoglobulin were made using paired non-parametric tests (sign rank test, Stata8).

## 3 Results

### 3.1 Subjects and samples

Thirty-three volunteers were selected and all completed the study. None was a current or ex-smoker of cigarettes and none had any history of pulmonary disease. Demographic characteristics, data from FEV1 testing and the results of bronchoalveolar lavage are summarised in Table 1. There were no significant differences in the age, gender distribution or FEV1 of the groups. The group randomized to receive injected vaccine had a lower mean BAL recovery volume at both bronchoscopies than the group randomized to receive inhaled vaccine. There were no side effects of bronchoscopy in either group and adequate samples were recovered from all participants. The mean BAL recovery volume was lower in the second bronchoscopy than the first for both groups. In addition, the cell count was increased in second bronchoscopy compared to the first, consistent with a pro-inflammatory effect of bronchoscopy but the increment in BAL volume and cell count were not different between the injected and inhaled vaccine groups.

### 3.2 Side-effects of injected and inhaled vaccine

Twenty-eight subjects reported minor side-effects of vaccination but there were no serious adverse events in this study. Fourteen subjects receiving subcutaneous vaccine reported side-effects including pain at the injection site (*n* = 9, of whom two required hydrocortisone cream), general malaise (*n* = 3) and headache. Twelve subjects receiving inhaled vaccine reported side-effects including dry mouth, distended abdomen, headache, diarrhea, dizziness and chills. Two subjects reported injection site pain after placebo injection.

### 3.3 Nebuliser output

The nebuliser produced a range of particle sizes in a normal distribution with a median aerosol diameter (MMAD) of 2 μm (2.3 μm and 1.8 μm in two trials with the distribution shown in detail in the on-line supplement). This particle size is consistent with alveolar deposition. Using a capture ELISA method (calibration curve *R*^2^ = 0.98), measurements of post-nebulised fractions were shown to have the same concentration of polysaccharide as new vaccine. 23-PPV concentration was measured after elution of impact filters from 0.5 ml of nebulised vaccine. The quantity of vaccine in the top and next two fractions (very small particle size) was too small to measure. The next four fractions (these sizes are respirable and deposited in alveoli) all contained detectable vaccine estimated at 0.026, 0.073, 0.116 and 0.0123 ml of vaccine. We therefore concluded that the nebuliser delivered respirable vaccine at a particle size consistent with alveolar deposition.

### 3.4 Serum IgG and IgA response to injected and inhaled 23-PPV

**Fig. 1:**
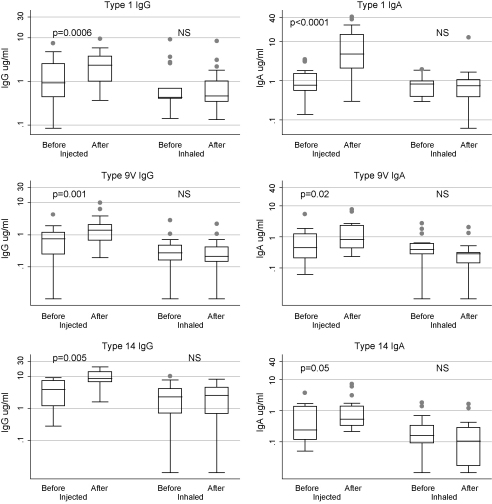
Comparison of serum responses to injected and inhaled vaccination with 23-valent pneumococcal capsular polysaccharide vaccine. IgG (left hand graphs) and IgA (right hand graphs) specific to pneumococcal capsular types 1, 9V and 14 are shown before and 1 month after vaccine. The box (25–75%) and whisker (5–95%) graphs with outlying values show median values for each group on a log scale. Following injected vaccine, a significant increase in serum IgG to all three capsular polysaccharide types tested was seen at 1 month. There was a significant increase seen in type 1 IgA only. Following inhaled vaccine, there was no significant increase in any serum parameter but there was a significant drop in both IgG and IgA to type 9V.

Fig. 1 shows serum values for IgG (left hand graphs) and IgA (right hand graphs) specific to pneumococcal capsular types 1, 9V and 14 tested at baseline and 1 month after vaccine. The box graphs show median values for each group on a log scale plotted by vaccine group before and after 1 month. Following injected vaccine, a significant increase in serum IgG to all 3 capsular polysaccharide types tested was seen at 1 month. There was a significant increase seen in type 1 IgA only.

Following inhaled vaccine, there was no significant increase in any serum parameter but there was a drop in both IgG and IgA to type 9V. Baseline values for serum immunoglobulin levels in the injected and inhaled vaccine groups were equal as shown in Table 2.

### 3.5 BAL IgG and IgA response to injected and inhaled 23-PPV

**Fig. 2:**
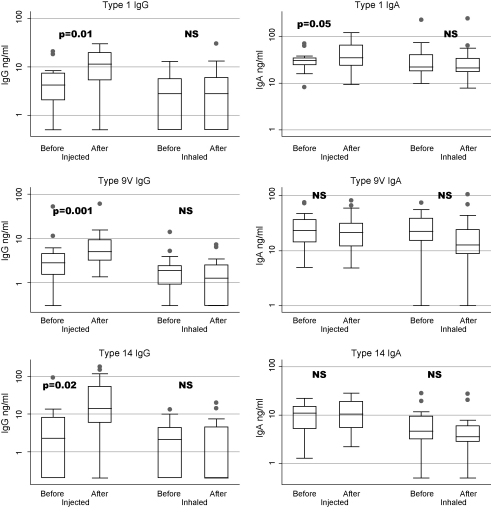
Comparison of bronchoalveolar lavage (BAL) responses to injected and inhaled vaccination with 23-valent pneumococcal capsular polysaccharide vaccine. IgG (left hand graphs) and IgA (right hand graphs) specific to pneumococcal capsular types 1, 9V and 14 were tested in BAL at baseline and 1 month after vaccine. The box (25–75%) and whisker (5–95%) graphs with outlying values show median values for each group on a log scale. Following injected vaccine, a significant increase in BAL IgG to all three capsular polysaccharide types tested was seen at 1 month. There was no significant increase in BAL IgA. Following inhaled vaccine, there was no significant increase in any serum parameter but there was a significant drop in BAL IgA to type 9V.

Fig. 2 shows BAL values for IgG (left hand graphs) and IgA (right hand graphs) specific to pneumococcal capsular types 1, 9V and 14 tested at baseline and 1 month after vaccine. The box graphs show median values for each group on a log scale plotted by vaccine group before and after 1 month. Following injected vaccine, a significant increase in BAL IgG to all three capsular polysaccharide types tested was seen at 1 month. There was no significant increase in BAL IgA.

Following inhaled vaccine, there was no significant increase in any serum parameter but there was a significant drop in BAL IgA to type 9V. Baseline values for BAL immunoglobulin levels in the injected and inhaled vaccine groups were equal as shown in Table 2.

## 4 Discussion

We compared the serum and bronchoalveolar lavageresponses to subcutaneous and inhaled 23-valent pneumococcal capsular polysaccharide (23-PPV) vaccination in healthy volunteers. The study showed that subcutaneous 23-PPV results in both BAL and serum immunoglobulin responses, albeit with lower responses in BAL fluid. Inhaled vaccine did not result in BAL or serum responses.

A methodological strength of this study is that serum and BAL were directly compared on the same ELISA plate in each subject before and after vaccination. In addition, three different vaccine capsular types recorded the same result. The serum results are consistent with published data on the response of healthy volunteers to subcutaneous or intramuscular 23-PPV [26,32]. The BAL data are novel and show that subcutaneous polysaccharide vaccination increases mucosal levels of IgG and IgA.

The results of our inhaled 23-PPV study are not completely consistent with other published data regarding the same dose of inhaled 23-PPV delivered to the alveolar space [26,27]. The studies do agree that inhaled 23-PPV is less effective than injected but in our series we did not see the increase in serum immunoglobulin levels in response to inhaled vaccine seen in some subjects (4/10 in healthy subjects, 7/10 in COPD patients) previous studies. There are several possible explanations for this discrepancy. The first is that the vaccine was not delivered to the alveolar space. We consider this unlikely because we have demonstrated that the vaccine was intact and in a respirable fraction and because the subjects experienced vaccine-related side-effects similar to those reported in previous studies [26]. The dataset available for comparison in this study was large enough (*n* = 17) to determine a range of values within the limits of sensitivity of the assays used and demonstrate the lack of response to inhaled vaccine with confidence as shown in Fig. 1. Previous studies reported either smaller numbers (*n* = 4 for serotype specific responses) or a pooled polysaccharide-specific response. One biological explanation is that our safety dose of 1/10 vaccine 24 h before the full dose may have blunted a possible response. We do not consider this likely as the duration is too short for a tolerising response and removal of relevant antibody may have had the opposite effect. Another biological explanation for the lack of response is that airway barrier functions including the mucociliary escalator and pre-existing antibody removed the vaccine from these healthy volunteers making it unavailable for absorption and systemic response or for local antibody response. Previous studies in COPD patients [27] compared to healthy controls [26] have shown higher total immunoglobulin responses in COPD consistent with increased absorption of vaccine through inflamed mucosa. Further, none of our subjects were cigarette smokers and smoke is known to increase allergen penetration through respiratory mucosa [33].

The first novel finding from this study is that subcutaneous 23-PPV results in increased immunoglobulin levels in BAL. 23-PPV is protective against invasive pneumococcal disease [34] but does not protect adults against pneumonia [17], and has been shown to cause an excess of pneumonia in some studies [15]. One explanation for these observations is that mucosal anti-capsular immunoglobulin may not be critical in protection against pneumonia. Indeed, there are published data to support the hypothesis that anti-pneumococcal capsular immunoglobulin, whilst being critical in defence against invasive pneumococcal disease, is not protective against mucosal infection. First, childhood clearance of nasal colonisation by pneumococci may be CD4 dependent and not dependent on capsular polysaccharide specific immunoglobulin [35]. Second, rather than being protective, pneumococcal type-specific anti-capsular IgA has been shown to be necessary for optimal pneumococcal colonisation of the nasopharynx by an IgA protease dependent mechanism [36]. Further, pneumococci colonizing the mucosa exhibit the transparent phenotype, in which the bacteria express relatively little capsule and have prominent surface proteins, in contrast to bacteria in the bloodstream which are predominantly heavily capsulated (opaque phase) [37,38]. We have reported that opsonisation alters binding and internalization of pneumococci by human alveolar macrophages, but the numbers of pneumococci observed to be ingested by alveolar macrophages in both our experiments [39] and those of others [40,41] were many orders of magnitude smaller than the ingestion observed in neutrophil experiments [42]. The alveolar macrophage may therefore be more of a sentinel than an active phagocyte in alveolar defence, and opsonisation may not have the critical significance that it has in the defence against invasive pneumococcal disease [43]. Finally, the observation that HIV infected adults with recent pneumococcal infection have higher BAL levels of anti-pneumococcal capsular immunoglobulin than HIV infected adults with no recent infection need not be interpreted as evidence of secondary protection. Higher levels of BAL anti-pneumococcal capsular immunoglobulin have also been measured in HIV infected adults than in healthy control subjects [10], and HIV infected adults have 20–100 times the incidence of pneumococcal disease seen in healthy adults [44]. In summary, therefore, it may be that BAL immunoglobulin is merely a marker of past disease, and of little protective use.

The second finding of this study was that inhaled 23-PPV did not result in enhanced immunoglobulin responses at the mucosal surface. The pulmonary mucosal surface is carefully regulated to avoid excess inflammation in response to inhaled antigens [45,46]. If anti-capsular immunoglobulin is not protective as discussed above, the lack of response observed in this study would be an appropriate immunological response. Upper airway responses were not assessed in this study but a comparison of upper and lower airway responses to pneumococcal carriage and vaccine antigens is in progress in our laboratory.

Animal studies have shown effective mucosal responses to whole cell vaccine and to some pneumococcal protein antigens but these studies have been confined to models where the upper airway architecture and defence is quite different to the human. In this study, the lack of local response to polysaccharide antigen is not all that surprising given that polysaccharide responses are normally initiated in the marginal zone of the human spleen.

Prevention of pneumococcal pneumonia remains an important global objective. A mucosal vaccine is attractive for the prevention of pneumonia as the known immunological compartmentalization of the pulmonary mucosa gives inhaled vaccination a plausible immunological basis in addition to the attractions of needle-free vaccination [47]. This study helps in the development of an inhaled vaccine in several ways. First, the importance of anti-capsular antibody at the mucosal surface has been questioned and is worthy of further study. Future inhaled vaccines should perhaps target the pneumococcal surface proteins, and in particular those that facilitate mucosal colonisation [48]. Second, this study has demonstrated that a safe and rigorous comparison of injected and inhaled vaccine was possible using BAL immunoglobulin as a marker of mucosal immunity.

## Figures and Tables

**Table 1:** Subject details by route of vaccination

Intervention	Injected vaccine	Inhaled vaccine	Difference, *p*
*N*	16	17	
Age in years (S.D.)	31.6 (12.2)	29.8 (8.1)	0.6
Gender (M:F)	11:5	13:4	0.6
Previous pneumonia or vaccine	0	0	
FEV1 in litres (S.D.)	2.7 (0.44)	3.1 (0.84)	0.1
BAL volume before vaccine in ml (S.D.)	118 (25.9)	136 (11.4)	0.01
BAL volume after vaccine in ml (S.D.)	112 (21.9)	128 (12.9)	0.01
BAL cells before vaccine (S.D.)	1.02 × 10^7^ (7.1 × 10^6^)	1.06 × 10^7^ (6.3 × 10^6^)	0.8
BAL cells after vaccine (S.D.)	2.81 × 10^7^ (1.5 × 10^7^)	2.59 × 10^7^ (1.1 × 10^7^)	0.65

**Table 2:** Geometric mean immunoglobulin levels with 95% confidence intervals

	Injected vaccine		Inhaled vaccine	
	Before	After 1 month	Before	After 1 month
Serum (μg/ml)				
Type 1 IgG	0.98 (0.53–1.82)	1.95 (1.2–3.2)	0.6 (0.38–1.17)	0.63 (0.36–1.1)
Type 1 IgA	0.88 (0.55–1.39)	5.4 (2.6–11.1)	0.69 (0.5–0.96)	0.67 (0.4–1.2)
Type 9V IgG	0.35 (0.1–1.3)	1.3 (0.7–2.4)	0.2 (0.07–0.6)	0.2 (0.06–0.5)
Type 9V IgA	0.5 (0.26–0.94)	0.97 (0.5–1.7)	0.35 (0.15–0.78)	0.2 (0.06–0.5)
Type 14 IgG	2.75 (1.5–5.0)	8.7 (6.3–12.0)	1.44 (0.56–3.67)	2.0 (0.9–4.0)
Type 14 IgA	0.32 (0.16–0.64)	0.74 (0.4–1.4)	0.23 (0.12–0.42)	0.17 (0.08–0.37)

BAL (ng/ml)				
Type 1 IgG	5.0 (3.2–7.9)	9.8 (5.9–16.3)	3.9 (2.3–7.0)	4.4 (2.3–8.4)
Type 1 IgA	28.6 (21.8–37.4)	35.9 (24.1–53.6)	27.6 (18.0–41.0)	25.5 (16.9–38.5)
Type 9V IgG	2.7 (1.4–5.5)	5.6 (3.3–9.4)	1.4 (0.7–2.6)	0.8 (0.4–1.7)
Type 9V IgA	22.7 (15.2–34)	20.1 (13.8–32)	17.5 (8.3–37.1)	11.9 (5.4–26.3)
Type 14 IgG	6.2 (2.5–15.4)	23.6 (10.8–51.3)	3.4 (1.6–7.2)	4.2 (1.4–12.3)
Type 14 IgA	8.2 (5.2–12.6)	9.6 (6.4–14.5)	5.6 (3.7–8.7)	4.3 (2.6–7.1)
