# Supplementary material for: Inhaled delivery of 23-valent pneumococcal polysaccharide vaccine does not result in enhanced pulmonary mucosal immunoglobulin responses
Source: Vaccine. 2008 Oct 3;26(42):5400–6. doi: 10.1016/j.vaccine.2008.07.082 (PMC2612086; doi:10.1016/j.vaccine.2008.07.082)

|  | Nebuliser testing results in ng/ml (concentration of NaF after filter soaked in 5ml) | | | | | | | |
| --- | --- | --- | --- | --- | --- | --- | --- | --- |
|  |  | Expt 1 | Expt 2 |  |  |  |  |  |
|  | top stage | 0.442 | 0.392 |  |  |  |  |  |
|  | 1 | 0.616 | 0.398 |  |  |  |  |  |
|  | 2 | 0.479 | 0.176 |  |  |  |  |  |
|  | 3 | 1.756 | 1.005 |  |  |  |  |  |
|  | 4 | 2.736 | 1.403 |  |  |  |  |  |
|  | 5 | 2.056 | 3.239 |  |  |  |  |  |
|  | 6 | 3.156 | 7.355 |  |  |  |  |  |
|  | 7 | 4.302 | 5.923 |  |  |  |  |  |
|  | 8 | 3.031 | 3.014 |  |  |  |  |  |
|  | final | 0.724 | 1.911 |  |  |  |  |  |
|  | Total | 19.298 | 24.816 |  |  |  |  |  |
|  |  |  |  |  |  |  |  |  |
|  |  |  |  |  |  |  |  |  |
|  |  |  |  |  |  |  |  |  |
| Expt 1 | ng/ml | Dlog (Dp) | conc/ | GMD (µm) | conc/ | Dp (µm) | %<Dp | V/Vtot (%) |
| GSD 3.5 |  |  | Dlog (Dp) |  | Dlog (Dp) |  |  |  |
| **MMAD 2.3** |  |  |  |  | normalised |  |  |  |
| Top | 0.442 |  |  |  |  | 50 | 97.70961 | 2.290393 |
| Stage 1 | 0.616 | 0.853316 | 0.72189 | 32.63434 | 2.1116 | 21.3 | 94.51757 | 3.192041 |
| Stage 2 | 0.479 | 0.36408 | 1.315645 | 17.755 | 3.848395 | 14.8 | 92.03544 | 2.482122 |
| Stage 3 | 1.756 | 0.412245 | 4.259605 | 12.04326 | 12.45977 | 9.8 | 82.93606 | 9.099389 |
| Stage 4 | 2.736 | 0.490623 | 5.576584 | 7.668116 | 16.31207 | 6 | 68.75842 | 14.17763 |
| Stage 5 | 2.056 | 0.538997 | 3.814496 | 4.582576 | 11.15778 | 3.5 | 58.10447 | 10.65395 |
| Stage 6 | 3.156 | 0.814508 | 3.874732 | 2.329163 | 11.33398 | 1.55 | 41.75044 | 16.35403 |
| Stage 7 | 4.302 | 0.510826 | 8.421661 | 1.200625 | 24.6342 | 0.93 | 19.45797 | 22.29247 |
| Stage 8 | 3.031 | 0.581356 | 5.213675 | 0.695414 | 15.25052 | 0.52 | 3.751684 | 15.70629 |
| final | 0.724 | 0.732368 | 0.988574 | 0.360555 | 2.891679 | 0.25 | 0 | 3.751684 |
|  | 19.30 |  | 34.18686 |  | 100 |  |  | 100 |
|  |  |  |  |  |  |  |  |  |
| Expt 2 | µl NaF | Dlog (Dp) | conc/ | GMD (µm) | conc/ | Dp (µm) | %<Dp | V/Vtot (%) |
| GSD 2.7 |  |  | Dlog (Dp) |  | Dlog (Dp) |  |  |  |
| **MMAD 1.8** |  |  |  |  | normalised |  |  |  |
| Top | 0.392 |  |  |  |  | 50 | 98.42037 | 1.579626 |
| Stage 1 | 0.398 | 0.853316 | 0.466416 | 32.63434 | 1.146679 | 21.3 | 96.81657 | 1.603804 |
| Stage 2 | 0.176 | 0.36408 | 0.48341 | 17.755 | 1.18846 | 14.8 | 96.10735 | 0.70922 |
| Stage 3 | 1.005 | 0.412245 | 2.437872 | 12.04326 | 5.993485 | 9.8 | 92.05754 | 4.049807 |
| Stage 4 | 1.403 | 0.490623 | 2.85963 | 7.668116 | 7.030373 | 6 | 86.40393 | 5.653611 |
| Stage 5 | 3.239 | 0.538997 | 6.009315 | 4.582576 | 14.77384 | 3.5 | 73.35187 | 13.05206 |
| Stage 6 | 7.355 | 0.814508 | 9.029991 | 2.329163 | 22.20015 | 1.55 | 43.71373 | 29.63814 |
| Stage 7 | 5.923 | 0.510826 | 11.59495 | 1.200625 | 28.50609 | 0.93 | 19.84607 | 23.86767 |
| Stage 8 | 3.014 | 0.581356 | 5.184433 | 0.695414 | 12.74588 | 0.52 | 7.700677 | 12.14539 |
| final | 1.911 | 0.732368 | 2.609344 | 0.360555 | 6.415048 | 0.25 | 0 | 7.700677 |
|  | 24.82 |  | 40.67537 |  | 100 | 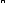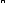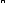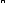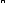   |  | | --- | |  | 100 |


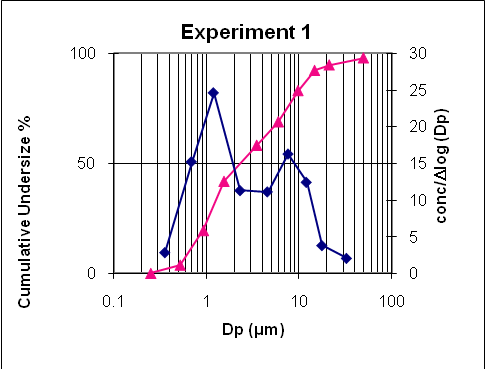


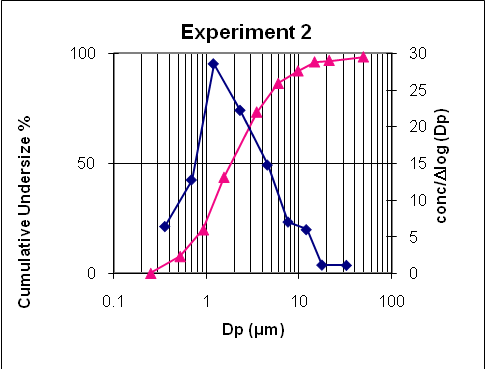

Supplement: Supplementary file 1 [file mmc1.doc]
